# Supplementary material for: Temperature Sensitivity of Topsoil Organic Matter Decomposition Does Not Depend on Vegetation Types in Mountains
Source: Plants (Basel). 2022 Oct 19;11(20):2765. doi: 10.3390/plants11202765 (PMC9611105; doi:10.3390/plants11202765)
Supplement: Supplementary file 1 [file plants-11-02765-s001.zip › plants-1874587-supplementary.pdf]

Supplementary materials

Table S1. Pearson's correlation coefficients between studied properties of long and short mountain transects with different land use (*n* = 33;**P* ≤*0.01*, ***0.001*).

Variable	Q_{10}	Altitude	Cover	Richness	C	pH	C:N	BR:C	MBC:C
Q_{10}	1.00								
Altitude	-0.07	1.00							
Cover	-0.20	*0.53**	1.00						
Richness	-0.38	0.28	*0.58***	1.00					
C	0.12	*0.68***	*0.66***	0.38	1.00				
pH	*0.45**	-0.01	-0.12	-0.28	-0.16	1.00			
C:N	0.36	-0.31	-0.44	-0.38	-0.19	0.41	1.00		
BR:C	*-0.51**	-0.19	0.09	0.09	-0.17	-0.14	0.03	1.00	
MBC:C	-0.14	0.40	0.27	0.25	0.07	0.07	-0.04	0.25	1.00
